# Gut microbiota and type 2 diabetes associations: a meta-analysis of 16S studies and their methodological challenges

**DOI:** 10.3389/frmbi.2025.1506387

**Published:** 2025-03-06

**Authors:** Jésica Lígia Picanço Machado, Ana Paula Schaan, Izabela Mamede, Gabriel Rocha Fernandes

**Affiliations:** ^1^ Biosystems Informatics and Genomics, Institute René Rachou, Belo Horizonte, Mina Gerais, Brazil; ^2^ Programa de Pós Graduação em Bioinformática, Institute of Biological Sciences, Universidade Federal de Minas Gerais, Belo Horizonte, Brazil; ^3^ Institute of Experimental Medicine, Kiel University and University Medical Center Schleswig-Holstein, Kiel, Germany; ^4^ Programa de Pós Graduação em Bioquímica e Imunologia, Institute of Biological Sciences, Universidade Federal de Minas Gerais, Belo Horizonte, Brazil; ^5^ Department of Biochemistry and Imunology, Institute of Biological Sciences, Universidade Federal de Minas Gerais, Belo Horizonte, Brazil

**Keywords:** gut microbiota, type 2 diabetes, methodology, meta-analysis, 16S

## Abstract

Diabetes mellitus is a prevalent chronic non-communicable disease, and recent studies have explored the link between gut microbiota and its development. Despite some evidence suggesting an association, the influence of gut microbiota on type 2 diabetes (T2D) remains unclear. A systematic search of PubMed (January 2016– December 2023) using the keywords “16S” and “diabetes” or “DM2” or “T2DM” or “T2D” and “gut microbiota” and “diabetes” or “DM2” or “T2DM” or “T2D”. The studies included compared gut microbiome diversity between diabetic and non-diabetic adults using 16S rRNA sequencing, excluding children, interventions, and type 1 diabetes. Alpha diversity indices and bacterial mean abundance were analyzed, with statistical assessments using a random-effects model and I^2^ for heterogeneity. Thirteen studies met the criteria, with the Shannon index being the most commonly used measure. Results showed significant heterogeneity (I^2^ > 75%) and no notable differences between diabetic and non-diabetic groups. Other indices, such as Chao1 and phylogenetic whole tree, similarly showed no consistent differences. Taxonomic analysis also failed to find phyla consistently correlated with T2D, with variability across studies. The relationship between gut microbiota and diabetes remains uncertain due to technical and biological factors that are often overlooked. The inconsistencies across studies highlight the low reproducibility common in microbiota research.

## 1 Introduction

As global populations increasingly adopt urbanized lifestyles, the prevalence of chronic non-communicable diseases, such as diabetes mellitus (DM), has become a significant public health concern, particularly in low- and middle-income countries (World Health Organization, 2023). Type 2 diabetes (T2D), which constitutes approximately 90% of all diabetes cases, is estimated to affect over 500 million adults worldwide, representing a substantial and increasingly significant economic burden (International Diabetes Federation, 2021; Ong et al., 2023). Beyond its impact on glucose regulation, T2D is a major risk factor for cardiovascular diseases, which remain the leading cause of death globally (World Health Organization, 2024).

T2D is a chronic condition marked by the reduced ability of the pancreas to produce insulin or the decreased effectiveness of insulin, leading to persistent hyperglycemia (World Health Organization, 2024). This multifactorial disease is influenced by genetic predisposition, environmental factors, and, more recently, alterations in the gut microbiome (Gilbert et al., 2018; Qin et al., 2012).

Numerous studies have proposed a role for the gut microbiome in the pathophysiology of T2D, attributed to its influence on host metabolic homeostasis. The gut microbiota contributes to maintaining the integrity of the epithelial barrier, maturing the immune system, and producing a variety of metabolites that exert systemic effects on the host (Bäckhed et al., 2012; Rogers and Wesselingh, 2016). Furthermore, reports have shown that microbial metabolization of dietary nutrients affects the energetic yield within the host, potentially contributing to the onset of obesity and pre-diabetes (Takeuchi et al., 2023). This process suggests a possible involvement of the microbiome in metabolic disorders by influencing insulin resistance and low-grade inflammation through the metabolism of dietary monosaccharides (Zhou et al., 2019).

The relationship between the gut microbiota and T2D, however, remains contentious, with inconsistent findings across different populations (He et al., 2018; Zhou et al., 2019). For instance, the genus Bacteroides has been reported to have both higher and lower relative abundance in diabetic patients across various studies (He et al., 2018; Yamaguchi et al., 2016). Some meta-analyses have highlighted this inconsistency, suggesting that the gut microbiome may not play a significant role in T2D development (Gurung et al., 2020; MetaHIT consortium et al., 2015). This has led to the hypothesis that it is the overall functional repertoire and metabolic output of the microbial community, rather than specific taxa, that are critical in the interaction between the microbiome and T2D Vatanen et al., 2018.

Concerns about the reproducibility of metagenomic studies, particularly in methodology, have also emerged. Notably, a highly cited article foundational to many studies was found to have methodological flaws (Gihawi et al., 2023). In response to these issues, we conducted a meta-analysis of datasets where gut microbiota, assessed through 16S rRNA gene sequencing, was studied in relation to Type 2 Diabetes Mellitus.

## 2 Methods

### 2.1 Study design

This systematic review and meta-analysis aimed to evaluate the relationship between the gut microbiome and type 2 diabetes mellitus (T2D) by analyzing 16S rRNA sequencing data. The study was designed to synthesize available evidence, identify patterns or discrepancies in the findings, and assess the reproducibility of results across different studies. Our approach followed the PRISMA (Preferred Reporting Items for Systematic Reviews and Meta-Analyses) guidelines to ensure a rigorous and transparent methodology.

### 2.2 Data search

A comprehensive and systematic search was conducted in PubMed to identify relevant studies published between January 2016 to December 2023. The search strategy combined Medical Subject Headings (MeSH) terms and keywords to capture all pertinent literature. The search string included the following terms: (“16S” AND “diabetes” OR “DM2” OR “T2DM” OR “T2D”) AND (“gut microbiota” AND “diabetes” OR “DM2” OR “T2DM” OR “T2D”). To ensure the quality and relevance of the data, only peer-reviewed articles published in English were considered. The search was complemented by manual screening of reference lists from selected studies to identify any additional relevant publications.

### 2.3 Study selection

The selection process involved a multi-step approach. Initially, titles and abstracts were screened to eliminate studies that clearly did not meet the inclusion criteria. Full-text reviews were then conducted for studies that appeared potentially eligible. Studies were included if they met the following criteria: (1) compared gut microbiome diversity between adult diabetic and non-diabetic populations; (2) employed 16S rRNA sequencing as the primary method for microbiome analysis; and (3) were published in English. Studies were excluded based on the following criteria: (1) studies involving pediatric populations, due to differences in microbiome composition; (2) studies relying solely on quantitative PCR (qPCR) for bacterial abundance, as this method lacks the depth of 16S rRNA sequencing; (3) studies employing shotgun sequencing, which differ significantly in methodology and scope from 16S studies; and (4) studies focusing primarily on inflammatory markers or other non-microbiome-related associations with diabetes. This rigorous selection process ensured that the included studies were comparable and relevant to the research question.

### 2.4 Data extraction and analysis

Data extraction was conducted meticulously from various sources within the studies, including text, tables, and figures. Key data points extracted included: authors, year of publication, sample size per group, 16S rRNA primer sequences, DNA extraction kits used, data availability (e.g., public repositories), country of origin of the study, inclusion and exclusion criteria, statistical methods employed, mean values of alpha diversity indices, mean values of bacterial abundance, and the choice of Operational Taxonomic Units (OTUs) versus Amplicon Sequence Variants (ASVs) for sequence classification.

For studies where data were presented in figures, values were extracted using PlotDigitizer (PlotDigitizer, 2024), an image processing software that allows accurate digitization of graphical data. The extracted data were then analyzed using a mean difference test to compare alpha diversity indices and bacterial abundance between diabetic and non-diabetic groups. Subgroup analyses were conducted based on the sequencing method used (OTU vs. ASV) to explore potential differences in findings related to methodological variations. A random-effects model was employed for meta-analysis, as recommended by Review Manager (Higgins and Green, 2011) version 5.4, to account for variability across studies. Statistical significance was determined at a p-value threshold of < 0.05. Heterogeneity among studies was assessed using the I^2^ statistic, with the following classifications: low (0%-40%), moderate (30%-60%), substantial (50%-90%), and considerable (75%-100%) (ibid.). All statistical analyses were performed using RStudio (RStudio Team, 2024), with R version 4.3.0 (R Core Team, 2024) and the ‘meta’ package version 7.0.0 (Schwarzer et al., 2015), ensuring reproducibility and transparency of the analytical process.

## 3 Results

### 3.1 Study characteristics

**Figure 1:**
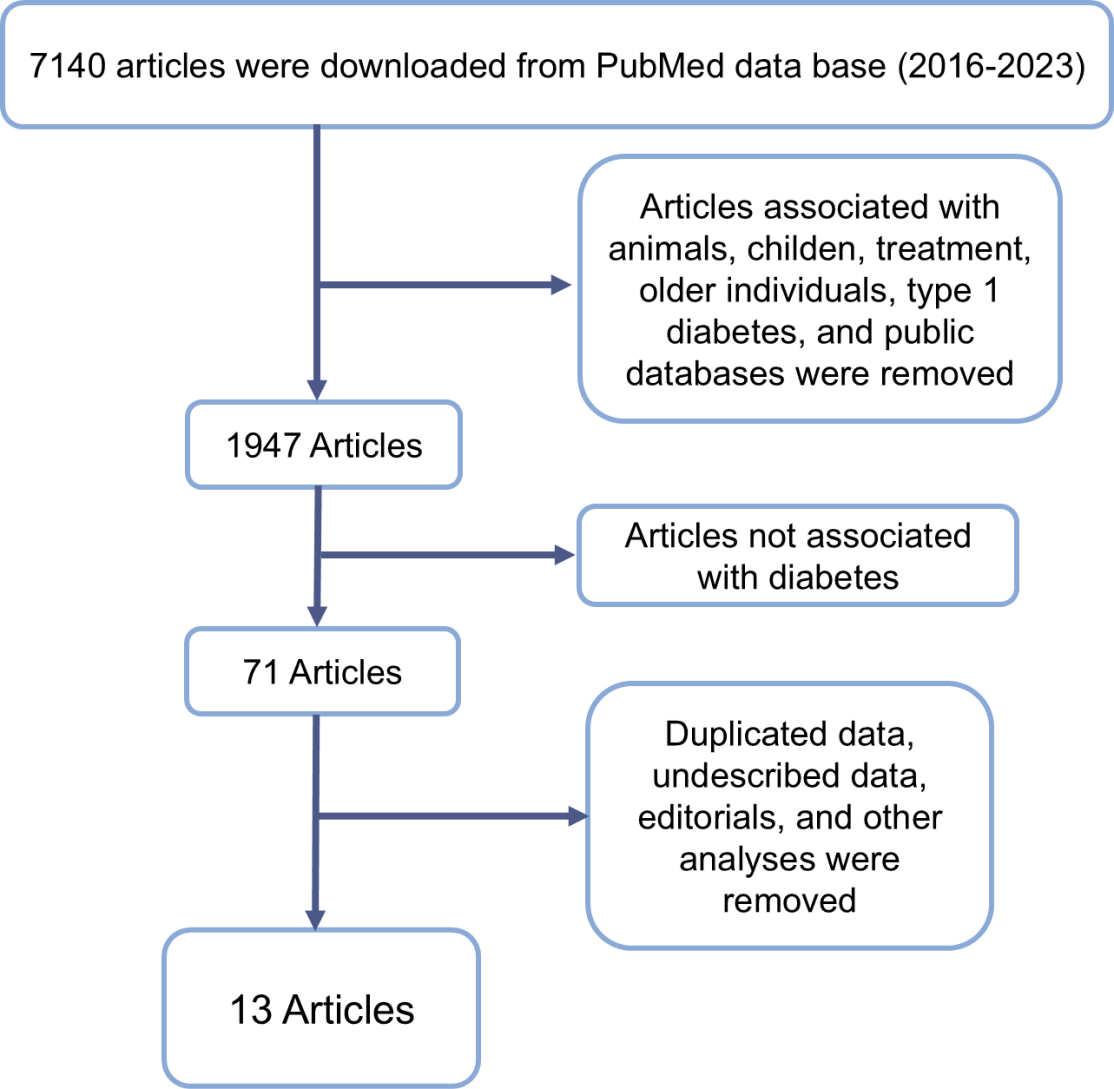
Flowchart representing inclusion and exclusion criteria and resulting article number after exclusion.

The initial search identified 7140 articles. After applying the inclusion criteria and narrowing down the results, 71 articles were selected for full-text review (**Figure 1**). Following this thorough screening process, thirteen studies met the criteria for inclusion in the final analysis. Of these, nine studies employed Operational Taxonomic Unit (OTU) sequences, while four utilized Amplicon Sequence Variants (ASV) for microbiome analysis.

Geographically, the majority of the studies were conducted in Asia, with eleven originating from this continent (China: 7, Japan: 1, Pakistan: 2). Two studies were conducted in North America (USA: 2), and one study was from the Middle East (Egypt: 1). Regarding taxonomic classification, the reference databases most frequently used were GreenGenes and SILVA, with each being utilized in four studies. The V3V4 region of the 16S rRNA gene was the most commonly targeted region for primer production, appearing in eight studies (**Table 1**). Across all included studies, a total of 4,066 sequenced samples were analyzed, providing a robust dataset for the meta-analysis.

**Table 1:** Study methodological characteristics.

Author	Country	Method	16S Region Reference	Database reference	N Size
Ahmad et al. (2019)	Paquistão	OTU	V3V4	Silva GreenGenes	60
Ding et al. (2023)	China	ASV	V3V4	GreenGenes2	101
Du et al. (2022)	China	OTU	V3V4	Not described	60
Guo et al. (2023)	China	ASV	V3V4	HOMD	168
Hashimoto et al. (2020)	Japão	ASV	V3V4	GreenGenes	194
Huang et al. (2023)	China	OTU	V4	GreenGenes	14
Li et al. (2020)	China	OTU	V4V5	GreenGenes	60
Maskarinec et al. (2021)	EUA	OTU	V1V3	SILVA	1702
Salah et al. (2019)	Egito	OTU	V3V4	SILVA	60
Saleem et al. (2022)	Paquistão	ASV	V3V4	SILVA	94
Walker et al. (2021)	EUA	OTU	V4	MetaPhlan2	1402
Wang et al. (2017)	China	OTU	V6	BlastN	40
Wang et al. (2020)	China	OTU	V3V4	RDB	171

**Figure 2:**
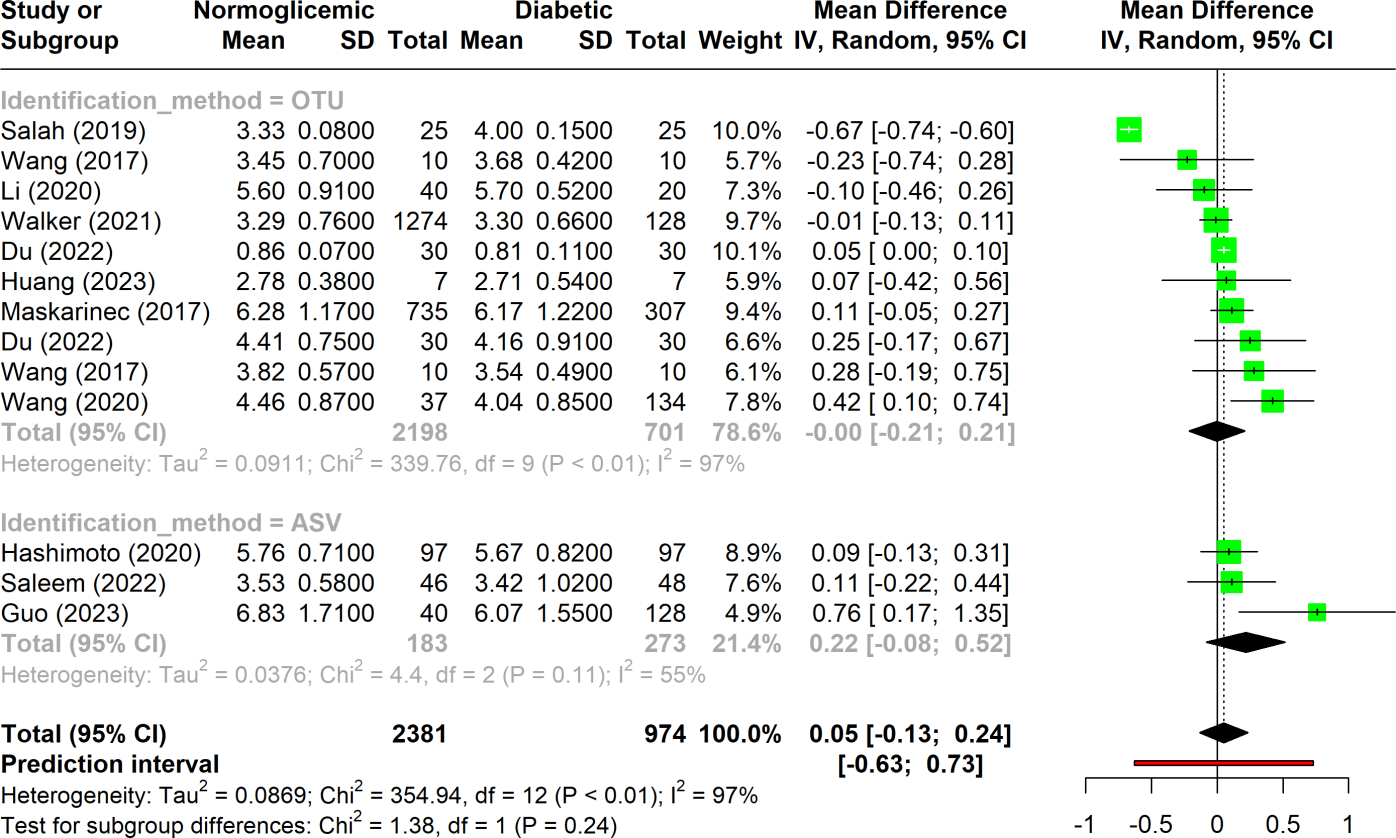
Forest plot of Shannon index in normoglycemic vs. diabetic subjects. Stratified by OTU and ASV identification methods.

The majority of the studies included in this meta-analysis (n = 11) utilized the Shannon index to evaluate alpha diversity between control and diabetic groups. As illustrated in **Figure 2**, there is substantial heterogeneity among the studies (I^2^ > 75%), suggesting that multiple factors contribute to the observed variability in alpha diversity results. This high heterogeneity indicates that the results are influenced by differences in study design, population characteristics, sequencing methods, or data analysis techniques.

Additionally, the results show considerable variation in the Shannon index across studies, as reflected by the wide confidence intervals and the non-significant p-value, which suggests no consistent difference in alpha diversity between diabetic and non-diabetic groups. When the studies were stratified into subgroups based on the identification method, it became evident that studies using the OTU approach exhibited greater heterogeneity compared to those employing the ASV method. The relatively low variability among ASV-based studies could be partly due to the smaller number of studies in this subgroup (only three), which may limit the generalizability of these findings.

**Figure 3:**
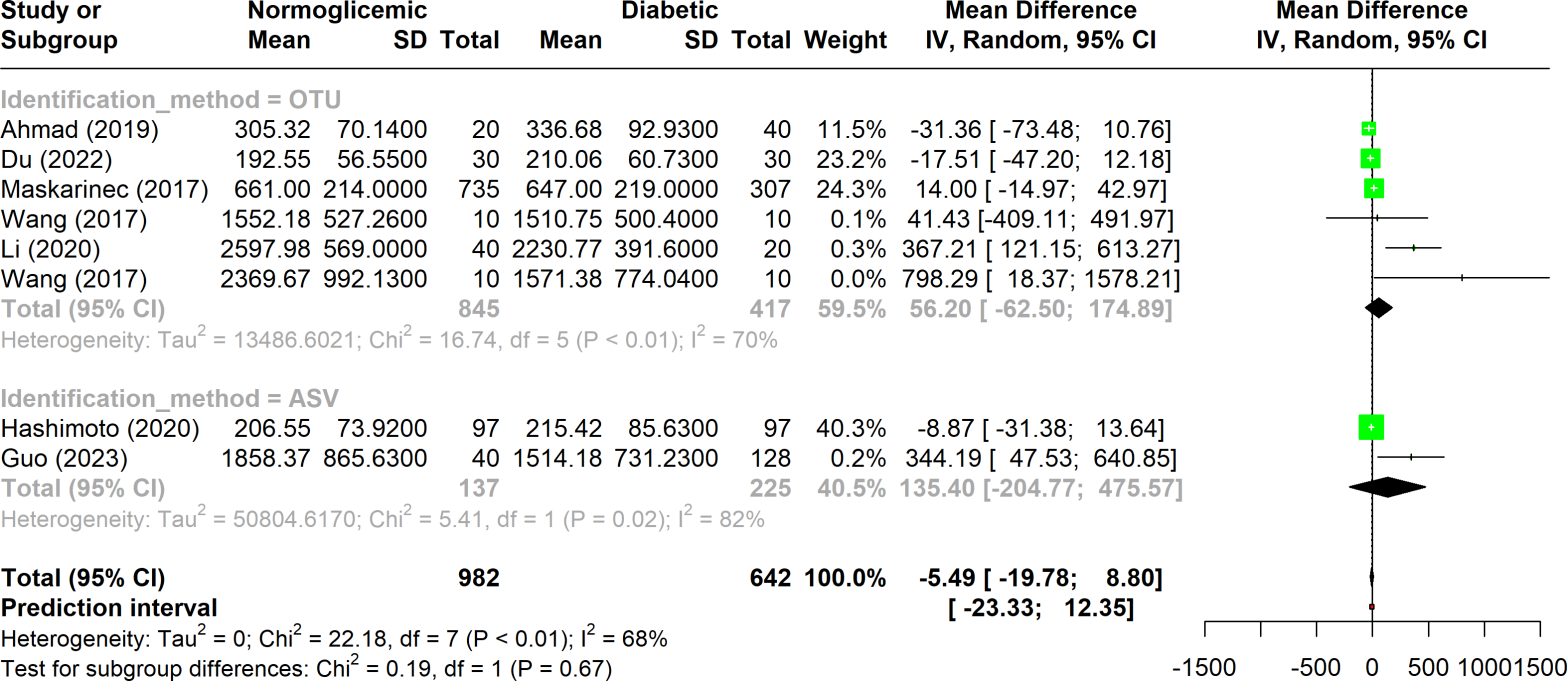
Forest plot of Chao1 index in normoglycemic vs. diabetic subjects. Stratified by OTU and ASV identification methods.

When the Chao1 index data from all included studies were analyzed using a forest plot, substantial heterogeneity was observed, with I^2^ values falling within the range of 50% to 75% (**Figure 3**). This suggests that while there is notable variability among studies, it is not extreme.

In four studies, higher alpha diversity was reported in diabetic individuals when OTUs were used for analysis. This trend was similarly observed in the ASV data, where two studies indicated an increased Chao1 index in diabetes, although the results were not consistent.

A specific subgroup of three studies that employed the OTU method exhibited substantial heterogeneity (I^2^ < 75%) and showed statistically significant variation (p-value < 0.01). However, despite this variation, no significant difference was found between the diabetic and non-diabetic groups within this subgroup.

**Figure 4:**
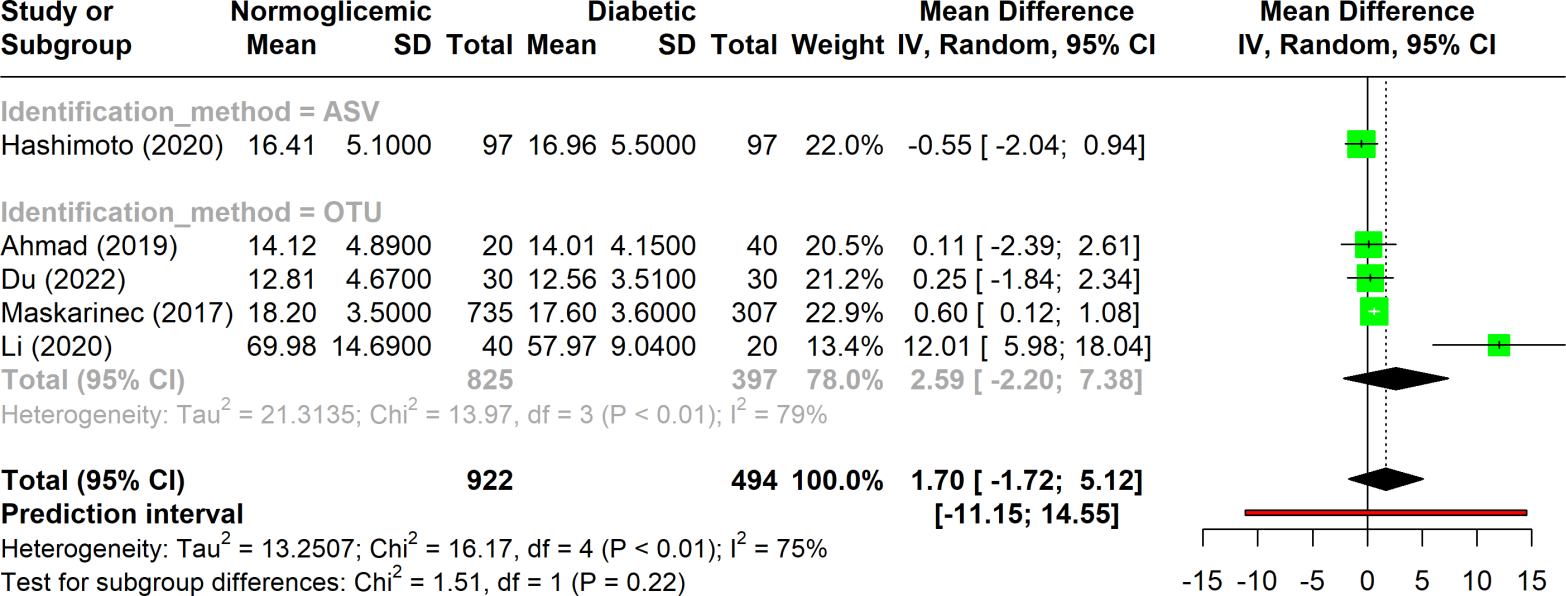
Forest plot of phylogenetic whole tree index in normoglycemic vs diabetic subjects. Stratified by OTU and ASV identification methods.

Five studies included in the analysis utilized the phylogenetic whole tree index to evaluate alpha diversity (**Figure 4**). This index showed substantial variation, with heterogeneity ranging from 50% to 90% (I^2^), suggesting notable variability across studies. Despite this, the overall p-value was significant (p < 0.01), indicating that there was no significant difference in phylogenetic distances between diabetic and non-diabetic groups. Among these studies, only one employed the ASV method, while the remaining four used the OTU method. The studies using the OTU method exhibited higher heterogeneity compared to the combined analysis of all five studies. Despite these methodological differences, none of the indices showed a significant difference in alpha diversity between the diabetic and non-diabetic groups.

### 3.2 Taxonomic composition

The analysis aimed to determine whether diabetic individuals have a distinct abundance of specific phyla compared to non-diabetic individuals. However, no clear trend was observed across the studies. Significant heterogeneity was evident (I^2^ > 75%), highlighting the diversity in the collected data. This variability suggests that the underlying factors contributing to differences in phylum abundance remain unclear and require further investigation.

**Figure 5:**
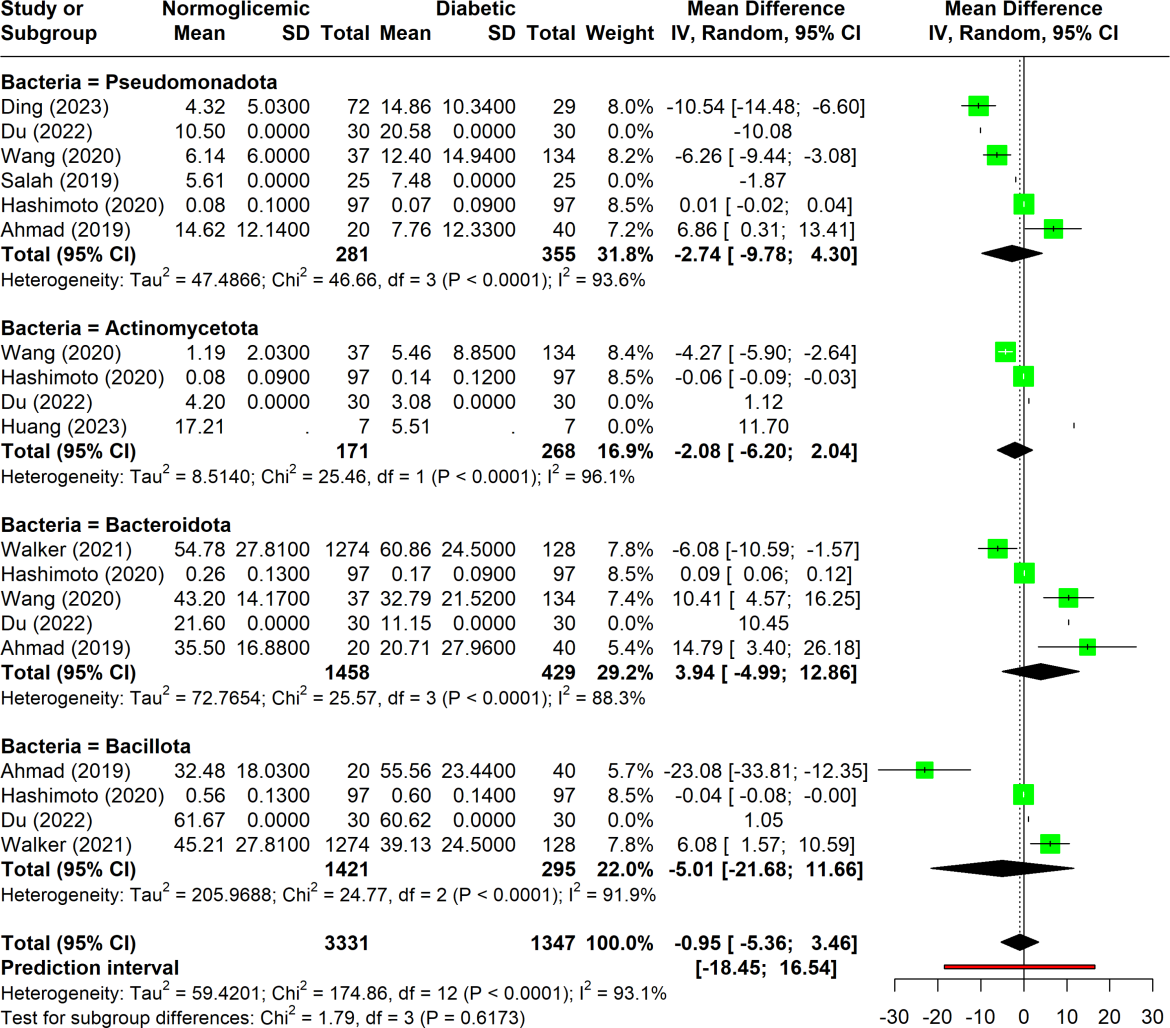
Forest plot of abundance of measurements among associated phyla.

Among the 13 studies analyzed, four phyla were frequently associated with diabetes, each showing considerable variation (I^2^ > 75%) and significant p-values (p < 0.05) (**Figure 5**). This disparity underscores the need for additional research to better understand these associations.

Pseudomonadota was the most commonly reported phylum, appearing in six studies. Despite its frequent mention, there was no consensus on its relationship with diabetes. The studies showed high heterogeneity (I^2^ = 94%), and no significant differences were found between normoglycemic and diabetic individuals concerning Pseudomonadota abundance. Furthermore, factors such as dietary habits and population characteristics, which may influence microbiota composition, have not been thoroughly investigated in this context.

Bacteroidota was the second most commonly associated phylum, mentioned in five studies. It was the only phylum with heterogeneity below 90%. The findings were mixed: Ahmad et al. (2019) and Du et al. (2022) reported higher Bacteroidota abundance in diabetic individuals, while Hashimoto et al. (2020) and Walker et al. (2021) found lower levels in diabetics. The confidence intervals and p-values suggest that there is no clear association between Bacteroidota abundance and diabetes.

Bacillota and Actinomycetota were each associated with diabetes in four studies. Bacillota exhibited a large confidence interval, mainly due to the findings in Ahmad et al. (2019) which indicated a significant difference in abundance between groups. However, the other three studies did not support this result. As for Actinomycetota, although slight variations in means were observed, the p-values and confidence intervals indicate no significant relationship between its abundance and diabetes.

## 4 Discussion

The association between complex traits such as Type 2 Diabetes Mellitus (T2D) and gut microbiota has been extensively proposed in the literature (Larsen et al., 2010; Baothman et al., 2016; Doumatey et al., 2020). However, our analysis reveals no significant differences in alpha diversity between normoglycemic and diabetic groups. This outcome is likely influenced by the substantial heterogeneity observed across the studies, suggesting that variations in results may be driven by multiple factors beyond microbial diversity, including methodological differences, personal eating habits, and population characteristics.

A key methodological factor is the choice between OTU and ASV approaches, with most studies favoring OTUs (n = 8). The OTU method, while common, is prone to replication issues due to its reliance on clustering algorithms, potentially merging different sequences into the same cluster. On the other hand, the ASV method, particularly when using the DADA2 workflow, offers more precise sequence identification through machine-learning algorithms and stricter merging criteria. Studies have shown that these methodological differences can lead to varying alpha diversity values even when analyzing the same dataset (Joos et al., 2020; Chiarello et al., 2022). Our results suggest that the lack of significant findings may stem from these methodological disparities, underscoring the need for standardized approaches in microbiome research.

Another critical factor is the sequencing depth, which can significantly impact alpha diversity indices. Indices like Shannon and Simpson’s are relatively robust, but Chao1, which was frequently used in these studies, is more sensitive to sequencing depth variations. This sensitivity might contribute to the observed variability, particularly when comparing OTU and ASV methods (Chiarello et al., 2022; Ramakodi, 2021). Additionally, the small sample sizes in most studies (10 to 40 individuals per group) may not accurately capture the true microbial diversity, introducing another layer of bias.

When evaluating taxonomic composition, our findings indicate no consistent differences in gut microbiota between diabetic and non-diabetic individuals, despite individual studies reporting differential abundances. The choice of reference databases, such as the outdated Greengenes (DeSantis et al., 2006; Bolyen et al., 2019) or the more recent Silva (Quast et al., 2012) can introduce significant variation in taxonomic identification, leading to inconsistent results. This lack of standardization highlights a major challenge in microbiome research, where the diversity of reference databases and methodological approaches creates noise and complicates the interpretation of findings.

The reported alterations in specific phyla, such as Pseudomonadota and Bacteroidota, also exhibit significant heterogeneity (I^2^ > 75%), suggesting that these findings are not reproducible across studies. For example, Proteobacteria, although frequently associated with diabetes, showed no consistent pattern of alteration, likely due to methodological differences and unconsidered confounding factors such as diet and population-specific characteristics. Similarly, Bacteroidota, despite being the second most commonly reported phylum, showed conflicting results across studies, further emphasizing the need for standardized methodologies.

The limited statistical power of alpha diversity indices in characterizing gut microbiota is another important consideration. The inherent inter-individual variability in gut microbiome studies necessitates larger sample sizes to achieve reliable assessments (He et al., 2018; Kers and Saccenti, 2022; Rothschild et al., 2018). Most studies analyzed here did not account for this variability adequately, leading to potential biases. The lack of consistent exclusion criteria, such as accounting for recent diarrhea or constipation, can further exacerbate the heterogeneity observed in microbial diversity and abundance (Vandeputte et al., 2016; Park et al., 2024).

Lifestyle factors, often overlooked in these studies, play a crucial role in shaping the gut microbiome. Recent evidence suggests that microbiota variations are more strongly associated with diet and environmental factors than with disease status alone (He et al., 2018; Trefflich et al., 2020; Gihawi et al., 2023). This perspective aligns with our findings, which indicate that diabetes alone is insufficient to explain the observed microbiota variation. Comprehensive analyses that consider multiple variables are essential for a more accurate understanding of microbiome dynamics.

Finally, the application of 16S rRNA sequencing to human samples presents unique challenges, as even minor environmental differences can lead to significant microbiome variations (Zuniga-Chaves et al., 2023). Detailed patient metadata, including dietary habits, stool consistency, and other health conditions, should be a standard inclusion in microbiome studies to improve the reproducibility and interpretability of results. Moreover, integrating metabolic biomarkers with microbiota data may offer more insights into diabetes-related variations than microbiota analysis alone (Yan et al., 2023; Gihawi et al., 2023).

In conclusion, the reproducibility issues observed in gut microbiota research related to Type 2 diabetes highlight the need for standardized methodologies, comprehensive biological data, and careful consideration of confounding factors. Addressing these challenges is crucial for advancing our understanding of the complex interplay between gut microbiota and metabolic diseases. Our analysis reveals that the observed inconsistencies across studies on gut microbiota and type 2 diabetes (T2D) are likely influenced by methodological differences, particularly in taxonomic identification and reference database selection. To enhance reproducibility in future research, it’s crucial to standardize methodologies and incorporate comprehensive patient metadata, including dietary habits and stool consistency. Additionally, applying advanced statistical techniques, such as bootstrapping, can simulate subpopulations and assess the consistency of findings across these subgroups, offering a more robust understanding of the microbiome’s role in T2D. By addressing these variables and adopting more rigorous statistical approaches, the field can move toward more reliable and reproducible results in microbiome research.

## Data Availability

The original contributions presented in the study are included in the article/supplementary material. Further inquiries can be directed to the corresponding author.
